# Bovine reproductive immunoinfertility: pathogenesis and immunotherapy

**DOI:** 10.3389/fvets.2023.1248604

**Published:** 2023-10-05

**Authors:** Vinod Kumar Gupta, Tushar Kumar Mohanty, Mukesh Bhakat, Raju Kumar Dewry, Rahul Katiyar, Dipti Nain, Nadeem Shah, Manisha Sethi, Rupali Rautela, Mahak Singh, Sourabh Deori

**Affiliations:** ^1^Artificial Breeding Research Centre (ABRC), ICAR-National Dairy Research Institute, Karnal, India; ^2^Division of Animal and Fisheries Sciences, ICAR Research Complex for NEH Region, Umiam, Meghalaya, India; ^3^CAR-Central Institute for Research on Buffaloes, Hisar, India; ^4^CAR Research Complex for NEH Region, Nagaland Centre, Medziphema, India

**Keywords:** cattle, endometritis, immunoinfertility, immunomodulators, LPS

## Abstract

Infertility is one of the primary factors for cattle reproduction in the present scenario. Reproduction-related immunoinfertility mainly involves immunization against the antigens related to reproductive hormones (LHRH, GnRH, Gonadal steroids, PGF2α and oxytocin), spermatozoa, seminal plasma and ovum. Anovulation, delayed ovulation, sperm immobilization, failure of fertilization, prolonged uterine involution, extended calving interval, prolonged post-partum estrus and reduced conception rate could be a result of immunoinfertility that occur due to the blockage of receptor site by antibodies formed against hormones, sperm and ovum. Immunoinfertility can be treated in the animal by giving sexual rest to females, by using various reproductive technologies such as *in-vitro* fertilization, gamete intra fallopian tube transfer, and intracytoplasmic sperm injection, sperm washing and by treating the animals with immunomodulators such as LPS, Oyster glycogen, etc. This review summarizes the different causes of bovine reproductive immunoinfertility and amelioration strategies to overcome it.

## Introduction

Infertility is the major problem in bovine reproduction in the dairy industry. There are several causes of infertility such as physiological, anatomical, nutritional and managemental that may be diagnosed, however most of the time immunoinfertility is unexplained and misdiagnosed (1, 2). Immunoinfertility refers to the condition where the immune system recognizes gametes (sperm and eggs) as foreign and launches an immune response against them, leading to difficulties in conception (1, 3). In the context of reproductive biotechnologies, such as *in vitro* fertilization (IVF), immunoinfertility can pose challenges. Immune responses against sperm and ovum can be due to the presence of antigens on the surface of these cells that the immune system recognizes as non-self. Immune reactions can hinder fertilization and embryo implantation, resulting in infertility. This is more common in cases where male and female cattle have genetic variations in their major histocompatibility complex (MHC) genes, which play a key role in immune recognition. To address immunoinfertility in reproductive biotechnologies, techniques like Intracytoplasmic Sperm Injection (ICSI) can be used. ICSI involves injecting a single sperm directly into an egg, bypassing the need for the sperm to navigate through the female reproductive tract, where it might encounter an immune response (4). Immunoinfertility has mainly involved immunization against antigens which have a relation with reproduction. These antigens could be related to reproductive hormones (LHRH, GnRH, Gonadal steroids, PGF_2α_ and oxytocin), spermatozoa, seminal plasma and ovum (5). Antibodies that form against these antigens can block the receptor site of the hormones, ovum and spermatozoa; and may lead to anovulation, delayed ovulation, failure of fertilization, early embryonic death, repeat breeding, prolonged inter-estrus interval, prolonged uterine involution, uterine infections like endometritis, extended calving interval, prolonged post-partum estrus and reduced conception rate that cause huge economic loss (6). Reproductive disorders particularly endometritis is one of the major reproductive problems challenging for bovines and antibiotic resistance is further complicating the problem. Immunomodulators have the potential to replace the use of antibiotics against endometritis (7). The present review aims to summarize the findings on immunoinfertility including its diagnosis, prevention, and control and to overcome its effect by various immunomodulators.

## Pathogenesis of immunoinfertility

### Immunoinfertility due to breach in blood-testis barrier

Immunoinfertility can occur either as autoimmunity in both males and females or isoimmunity in females (1). Autoimmunity arises when antibodies are produced against spermatozoa in the body of the female (8). Autoimmunity in males has been associated with isoimmunity in females (9). Immune tolerance for self-antigen is established in the neonatal period, hence the new developing antigens appearing on sperm surface during spermatogenesis immunogenic sequestration, behind the blood-testis barrier formed by tight junctions of Sertoli cells, prevents the generation of autoantibodies to sperm (1). However, once the blood-testis barrier is breached due to mechanical, infectious, inflammatory or degenerative changes, it results in exposure of immunogenic sperm antigens to the immune system of animals (10). Therefore, an immune response initiates, resulting in anti-inflammatory reaction and antisperm antibody formation. It has been reported that ova are less immunogenic as compared to spermatozoa (11).

### Uterine infection induced immunoinfertility

**Figure 1:**
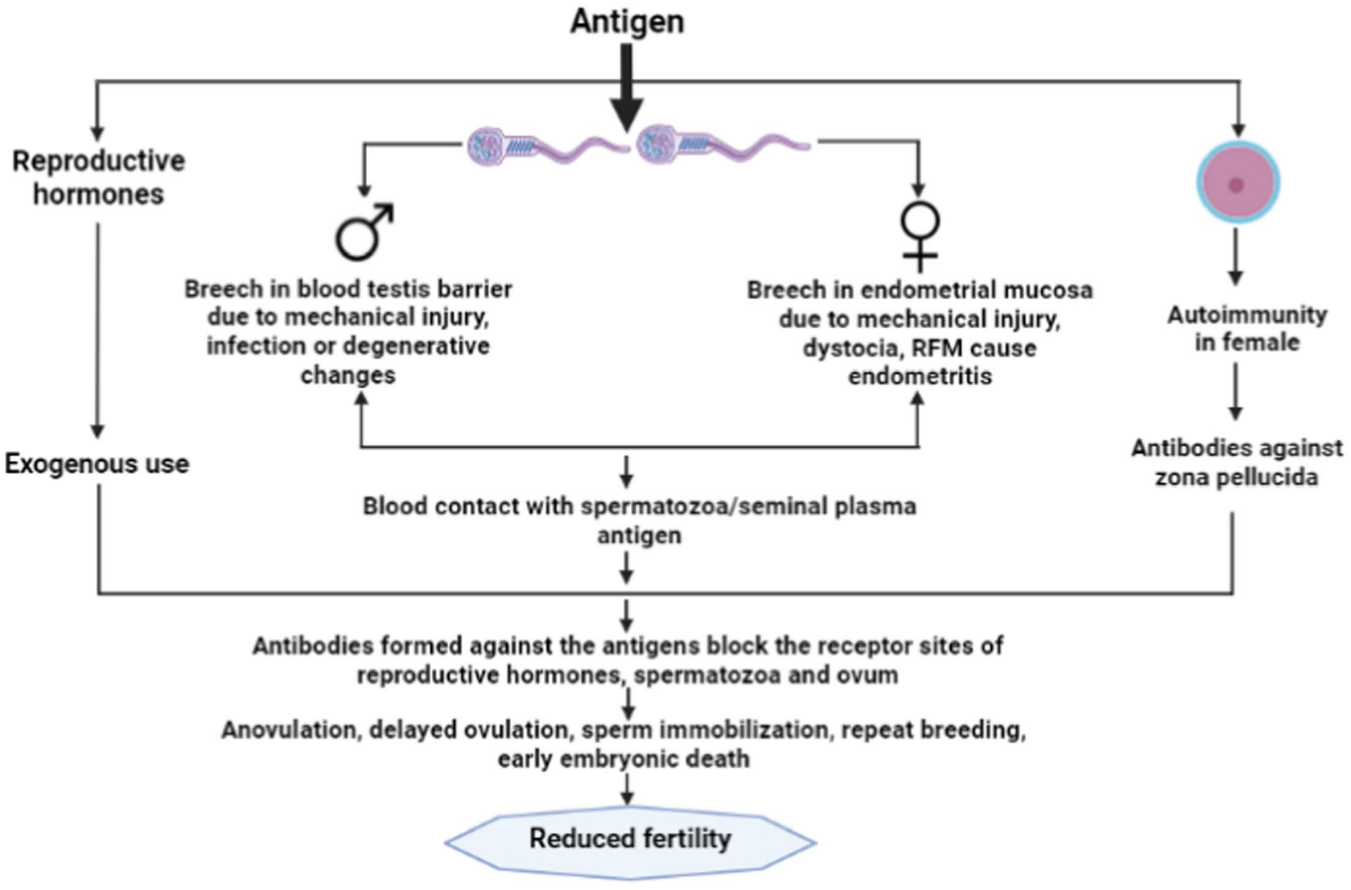
Immunoinfertility caused by exogenous use of reproductive hormones and autoimmunity in female.

Generally during natural mating female reproductive tract is exposed to spermatozoa, but it does not initiate the immune response against the spermatozoa due to the presence of immunoinhibitory substances like 19-hydroxy prostaglandin E, polyamines, transglutaminase and high-molecular-weight Fc receptor binding protein present in the seminal plasma, follicular and uterine fluid, as well as cervical mucus (1, 12) which may protect spermatozoa from immunogenic damage and prevent sensitization of a female to sperm antigens. However, in the case of endometritis which can occur due to any physical injury or bacterial infection in the mucosa of the endometrium, blood gets exposed to sperm or seminal plasma antigens at the time of artificial insemination or natural service (6). This exposure results into the formation of antisperm antibodies in the body of female animals and secreted in the cervical mucus and uterine fluid that could immobilize the spermatozoa (12). It has been reported that infection in the female reproductive tract particularly endometritis could play a potential role in immunoinfertility in female animals (13). Early postpartum dairy cows frequently experience uterine bacterial infections. Within the first 2 weeks after giving birth, 90% of cows suffer bacterial infections of the uterus (14). The two main pathogenic bacteria that are frequently seen to be connected to cow uterine infections are *Escherichia coli* and *Trueperella pyogenes* (14). The production of lipopolysacharide (LPS) by gram-negative bacteria, which is a major component of the cellular membrane, stimulates the innate immune system resulting in the inflammatory response (15). The LPS present in the peripheral circulation may translocate to follicular fluid resulting into ovulatory aberrations, however, the mechanism of translocation is still a topic of debate (16). The high LPS concentration in follicular fluid may disturb the transcription of transcription of gonadotropins. It has been observed that LPS impairs the follicular steroidogenesis act by direct action on theca and granulosa cells. Low estradiol levels in the follicular fluid and high caspase-3 mRNA expression in high-LPS follicles point to a connection with follicular atresia (17). Therefore, the immunity of uterus during the post-partum period plays an important role in the developmental competence of the oocyte (Figure 1).

## Types of antigens causing immunoinfertility

### Spermatozoal and seminal plasma antigens

Antigens on spermatozoa and seminal plasma play an important role in sperm function and conception. Seminal plasma antigens such as PH-20, PH-30, fertilization antigen-1 (18) albumin, globulin, glycoproteins, lactoferrin (19) and spermatozoal antigens like hyaluronidase, acrosin, neuraminidase and corona dispersing enzyme are the important antigen that helps in protein tyrosine phosphorylation, capacitation, acrosomal reaction, gamete fusion and fertilization. MHC1a and HY antigens are found on spermatozoa required for implantation of the embryo (20). IZUMO1 is a sperm surface protein that helps in capacitation and sperm oocyte fusion (21). Sperm surface proteins such as ADAM1, ADAM2 and ADAM3 have a potent role in sperm-oocyte binding (22). Several seminal plasma antigens are found in form of cytokines and antimicrobial peptides that regulate immune and inflammatory responses and maintain fertility (13). Beta defensin 126 is an antimicrobial peptide secreted from bovine epididymal epithelium found in semen and is associated with sperm motility, chemoattractant, cell signaling, sperm-oviductal epithelium binding and sperm-oocyte binding (23). It is required for the maturation of sperm in the male epididymis and the protection of sperm in the female reproductive tract (13). Antibodies formed against these antigens may impede its functionality and hamper sperm motility.

### Antigens of ova

The antigens of the zona pellucida in mammals are of special interest because of their possible involvement in immunoinfertility and as a candidate target for immune-contraception. The zona pellucida of the egg comprises three biochemically and immunologically distinct glycoproteins, i.e., ZP1, ZP2 and ZP3 of which spermatozoa bind to ZP3. Blocking the function of protein with antibodies results in infertility and this has been experimentally demonstrated in the horse, and monkeys (5, 24). Therefore ZP3 can be considered an important protein target for immune-contraception in the horse, and monkeys (24) and is being used presently for contraceptive vaccine production.

### Antigens in semen extenders

The important component of the semen extender is egg yolk and proteins of egg yolk may act as antigens, which was confirmed by isolating antibodies against the egg yolk antigens in inseminated heifers in the early postpartum period (25) as well as in vagina and uterus of inseminated cows (26).

### Antibodies and infertility

The impacts of the anti-sperm antibodies on infertility are concentration-dependent. Low and moderate concentrations of sperm antibodies in the blood do not affect the reproductive function of cattle while high concentrations are usually associated with infertility (27). Repeat breeder cows showed a titer of 1:512 when tested with the bulls which were previously used for inseminations, while conceived cows showed a titer of 1:16 or less in the first service (25). Frequent exposure to seminal antigens through repeated insemination elevates the antibody titer and results in lowered fertility and an increased number of inseminations per conception. The sperm antibody titer in the blood serum of cow does not exceed 1:16 when conception occurred but repeated services led to a rise in titer to 1:512. The presence of antibodies was confirmed by multiple means (25).

### Antisperm antibodies

**Figure 2:**
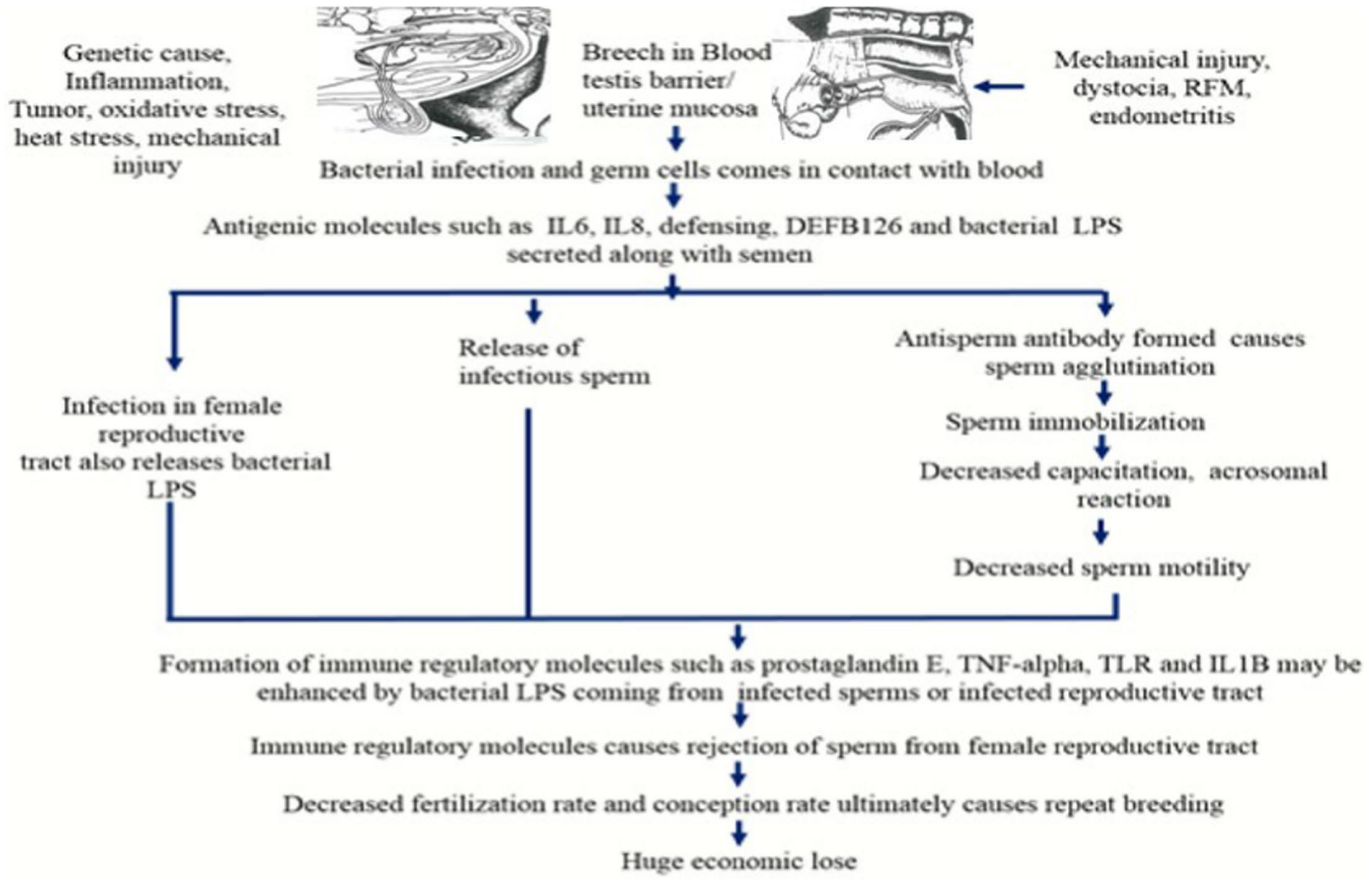
Antisperm antibody production by the immune system and its impact on sperm motility and fertility (RFM, Retension of fetal membrane; IL6, Interleukin 6; IL8, Interleukin 8; LPS, Lipopolysaccharide; TNF-alpha, Tumor necrotic factor-alpha; TLR, Toll like receptor; IL1B, Interleukin 1B).

The growing germ cells express new surface antigens during spermatogenesis, although these are difficult to distinguish. Surface antigens unique to sperm initially arise on the pachytene primary spermatocyte (28). When these sperm-specific surface antigens come into contact with blood at the time of surgical trauma, various microbial diseases such as prostitis and orchitis, testicular cancer, and varicocele, the formation of ASA can be accelerated. Antisperm antibodies can be found in a bull with orchitis 18 months after the initial manifestation. This represents the long effects of genital infections on fertility (29). The location, regional specificity and antigen specificity of Antisperm antibodies define their effects on fertility. Antisperm antibodies were found in seminal plasma or serum in the unbound form, however only the sperm bound Antisperm antibodies were found to have a detrimental effect. Sardoy et al. (11) found that immunoglobulins such as IgA and IgG, but not IgM, have a deleterious influence on fertility. The major reasons for infertility associated with antisperm antibodies are prevention of fertilization or early embryonic mortality. The possible mechanism involves in sperm antibody-mediated fertilization failure are sperm immobilization, inhibition of migration through the female genital tract, inactivation of acrosomal enzymes for fertilization, inhibition of sperm attachment and penetration to zona pellucida of ova and embryonic death. The ASA formation is induced by the effects on the fertilizing sperm and by actions on developing conceptus (11). Antisperm antibodies can also influence the peri-implantation embryo’s survival. Antigens produced from sperm may remain on the oolemma after fertilization. Due to the production of embryonic antigens that might cross-react with spermatozoa, antisperm antibodies may be produced during the early embryonic stage. These antibodies can cause activated cells in the female immune system to produce cytotoxic lymphokines, which can impact fetal development indirectly (30) (Figure 2).

## Economic consequences of immunoinfertility

Immunodeficiency in farm cattle can have significant economic consequences. When cattle have weakened immune systems, they are more susceptible to various reproductive diseases, leading to lower recovery, lower conception rate, increased mortality rates and reduced overall productivity. This results in higher veterinary expenses, as well as losses due to decreased milk production. Additionally, immunodeficiency might necessitate the use of antibiotics and other treatments, further increasing costs. Disease outbreaks can lead to trade restrictions and bans on cattle exports, affecting the entire industry. Consumer confidence can also be undermined, leading to decreased demand for cattle products (31). Overall, maintaining the immune health of farm cattle is crucial for minimizing economic losses and sustaining a profitable agricultural sector. Therefore, the diagnosis of causative factors involved in the immunoinfertility becomes essential.

### Diagnosis of antisperm antibody

ASA can be detected by direct and indirect tests. Indirect tests include Sperm agglutination test, Sperm immobilization test, Immuno-bead assay, ELISA, while direct test includes mixed agglutination reaction, Immunoflouroscence assay, Radiolabeled antiglobulin assay, flow cytometry etc. Preventive measures to reduce immunoinfertility include sexual rest, change of male and sero therapy (32). It was observed that cows had high antibody titers against seminal antigens of bulls which were commonly used for breeding purposes as compared to bulls which were either less frequently used or showed low titers against seminal antigens. In a study, a group of nine repeat breeders with high ASA titers were treated with a change of male, and six animals were found conceived (32) (Table 1).

**Table 1:** Table showing different diagnostic test to detect Antisperm antibodies and their principal in various breed/species.

Test	Principle	Species/Breed	References
Sperm agglutination test	Number of clumps with spermatozoa can be seen under the microscope after incubation of semen sample (60 million/ml) with test serum	Cattle serum	(6)
Sperm immobilization test	Guinea pig serum is used as a complement to immobilize the spermatozoa	Cattle serum/Seminal plasma	(6, 33)
Immunofluorescence test	Fluorescence-labeled secondary antibody is used to detect the Antisperm antibodies	Bull serum	(11)
Immuno-peroxidase test	–	Bull semen	(33)
Mixed antiglobulin reaction	Mixed antiglobulin reaction can detect surface antigens. Based on the coombs test. Mixed clumps of red blood cells and spermatozoa can be seen after agglutination with a slow “shaky” movement under a light microscope. Takes 10 min to perform	Human serum	(11)
Flowcytometry	With the use of calibration standards dead sperm from washed and stained samples are excluded with fluorescein-isothiocyanate-conjugated F(ab’)2 fragments of anti-IgG and IgA antibodies Sperm-bound antibodies can be quantitated and detected by flow cytometry	Bull serum	(29)
Immunobead-binding test	Antibody coated latex beads are used to know the localization, proportion and class of antibody attached to spermatozoa < 30 min takes to perform the test	Human serum	(11)
ELISA	ELISA combines the specificity of the antigen–antibody reaction with the continuous degradation of the chromogenic substrate by an enzyme to amplify the sensitivity of the reaction	Bull serum	(11, 34)

### Treatment of immunoinfertility

Several strategies can be used to treat anti-sperm antibody-mediated infertility. Immunodepletion, washing of sperm, and treatment with IgA protease are certain methods for the removal of Anti-sperm antibodies from the sperms (35). Anti-sperm antibodies in sperm samples can be isolated by coating magnetic microbeads with anti-immunoglobulin and combining them with sperm. The semen samples are then subjected to a magnetic cell sorter and the ASA positive sperm are removed from the sample (36). A significant reduction has been observed in the number of Anti-sperm antibody-bound sperm by applying this process. The use of such a process in assisted reproductive techniques like intrauterine insemination, intracervical insemination, *in-vitro* fertilization, gamete intrafallopian tube transfer, subzonal sperm injection, and intracytoplasmic sperm injection reduces the chances of gametes being exposed to anti-sperm antibody, which improves gamete function (37). Treatment of sperms with protease or chymotrypsin before intra-uterine insemination can reduce the Antisperm antibodies to sperm surface (37). Higher concentration of seminal zinc is found beneficial for sperm motility because it plays an important role in the prostate, epididymal and testicular functions and generates energy for sperm motility through lipid catabolism (38). Supplementation of zinc sulfate and zinc propionate to crossbred cattle bulls in their diet has improved the quality of semen (39).

### Uterine infection and infertility

Anatomically the endometrial gland mucus discharges; pseudostratified columnar epithelium covering the endometrium and immunologically, polymorphonuclear inflammatory cells (PMNs) and humoral antibodies (40). When these systems are disrupted, opportunistic infections, primarily microbes present in the posterior gastro-intestinal tract and surrounding the perineal area, can invade the endometrium and cause endometritis (41). Coitus, AI or more commonly, parturition can cause endometrial inflammation in cattle. The majority of cattle have microorganisms in their uterine lumen 1–4 weeks after calving, although they normally self-cure within 6 weeks. Cows that are unable to counter the infection may develop endometritis. A diagnosis is usually made during a routine check of cows after calving or when they are bred. Endometritis causes the animal’s conception to be delayed significantly (42).

The uterus of a postpartum cow has produced a wide range of bacteria, including Gram-positive and Gram-negative aerobes and anaerobes (43). *Arcanobacter pyogenes* is the most commonly isolated bacteria. (44). Other bacteria have been grown and associated with endometritis of varying severity, including *Streptococci, Staphylococci,* and *Escherchia coli* (45). Because both pathogenic and non-pathogenic organisms inhabit the bovine endometrium, and many of them are selective, it is been difficult to pinpoint the most important bacteria associated with endometritis (46). *Fusobacterium necrophorum* and *Bacteroides melaninogenicus* have been identified in the anaerobes cultured from cases of endometritis. Likely, they act synergistically with *A. pyogenes* in severe endometritis (47). An endometrial biopsy or a uterine discharge culture can easily identify endometritis (48, 49), however, these strategies cannot be used as a routine field screening tool to identify all cows in need of treatment. Haptoglobin in the peripheral blood has been used as a marker for endometritis by certain researchers. It is an acute-phase protein produced in the liver in response to tissue damage, and its primary function is to bind free hemoglobin and protect the host from the oxidative activity of hemoglobin (50).

### Immune mechanism involved in immunomodulation of uterine infection

The microbe is phagocytized by polymorph nuclear inflammatory cells (PMNs), blood monocytes, and tissue macrophages through various procedures such as chemotaxis, adhesion, and attachment of PMNs to cell surface antigens displayed by the pathogen (51). During normal unassisted calving, after 48 h not only the leukocytes but also the contaminant micro-organisms accumulate in the uterine lumen. This indicates the initiation of a normal uterine cleansing and involution process (52). The phagocytic activity of uterine PMN cells initially decreases during the puerperal metritis in bovines, however, the phagocytic activity of uterine PMN cells increases after 2–3 weeks, (53).

Neutrophil phagocytic activities are also influenced by hormonal fluctuations. Increased blood progesterone or cortisol levels, for example, lower neutrophil phagocytic activity in the uterus and peripheral circulation (54). The number of neutrophils in the peripheral blood increased steadily from about 6 weeks before parturition to a peak on the day of calving, although maternal and fetal cortisol may have a limited neutrophil function during this time (55). Shortly after calving, blood neutrophils’ phagocytic activity decreases, although the uterine lumen’s cellular defense mechanisms are maintained by increasing PMN levels (56). The quantity of PMN cells in the peripheral blood decreases during the first 1–3 weeks after calving, most likely due to the migration of PMN cells into the mammary gland and uterine lumen. The phagocytic activity of PMN cells decreased significantly in aged cows compared to younger ones (57). Both in cattle as well as in mare, the phagocytic activity of neutrophils was found higher in peripheral blood than in the uterine lumen (56). When known pathogenic micro-organisms were experimentally inoculated intrauterine, the order of emergence of immunoglobulins in cervical and vaginal secretions was IgM, IgA, and IgG, while the order of elimination of immunoglobulins was IgM, IgG, and IgA (58).

**Figure 3:**
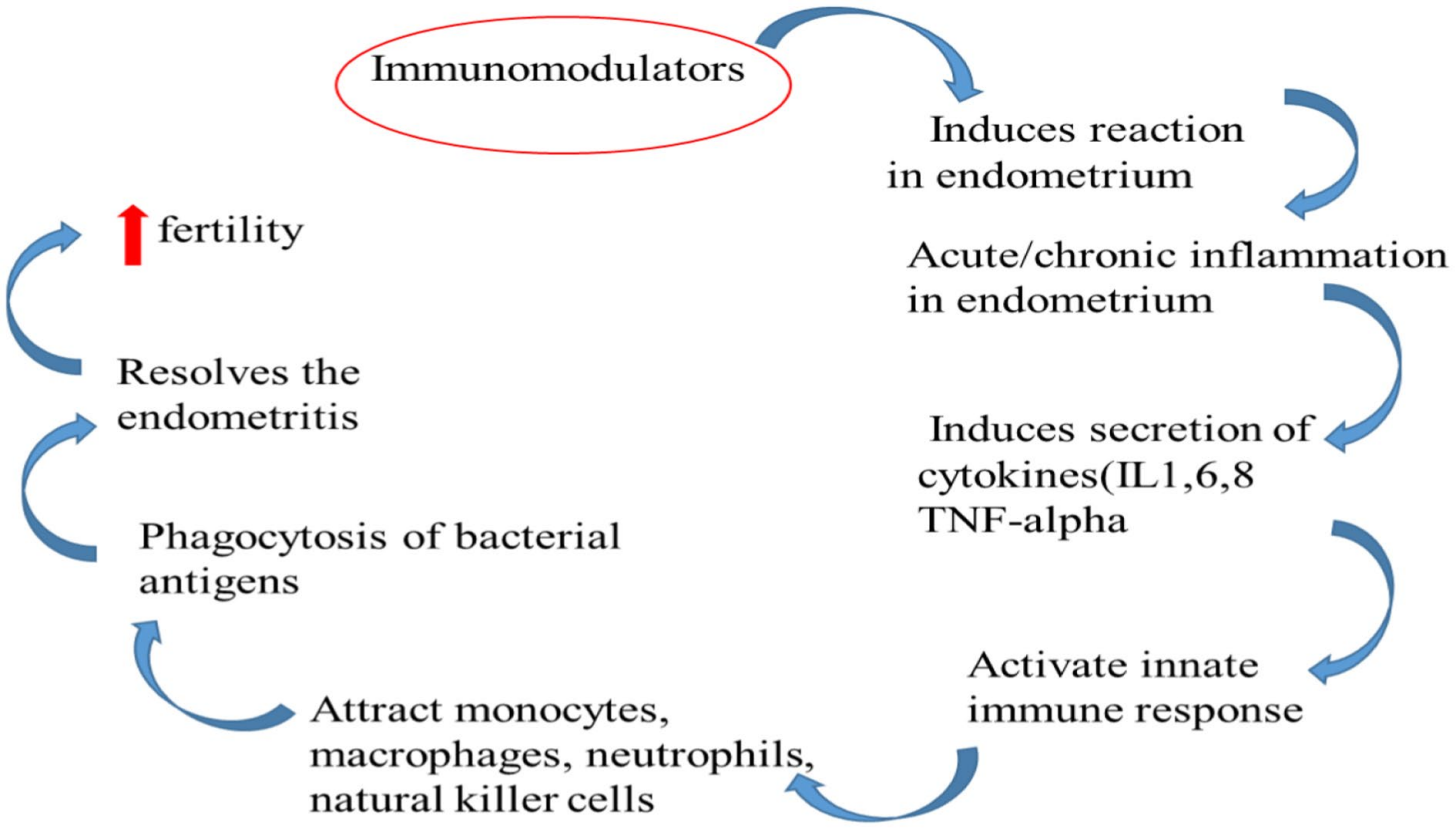
Immune mechanism made by immunomodulators to restore fertility.

IgG predominates in the uterine lumen, while IgA predominates in the vaginal canal (59). Although IgG concentrations in vaginal secretions are highest during oestrus (60), few investigations have looked into the specific influence of steroid hormones on immunoglobulin concentrations. The quantities of IgG and IgM in lochia from healthy cows drop after calving (61). Those cows having abnormal puerperium the concentration of immunoglobulins like IgA and IgG increases in uterine secretion as endometritis develops (62). IgA is made at the mucosal surface of the bovine uterus. The endometrium produces half of the IgG1 fraction, while the balance of the IgG1 and all of the IgG2 come from the peripheral circulation (63) (Figure 3).

### Immunomodulators as a replacement therapeutic for antibiotics

Several antibiotic therapies commonly used to treat postpartum problems in cattle may have a negative impact on the uterine cellular immune response (64). Manual removal of fetal membranes, for example, may reduce uterine leukocyte phagocytic activity for several days (65), as intrauterine administration of most antiseptics and disinfectants (51). Antibiotics administered intrauterine reduce uterine leukocytic activity, which could have major consequences in the treatment of metritis if the bacteria involved grow resistant to the antibiotic. Extensive use of antibiotics not only causes antimicrobial drug resistance but is also responsible for the secretion of drug residues in the milk (7). Apart from antibiotics, endometrial necrosis and fibrosis can be caused by overdose or continuous use of a variety of different drugs, such as Lugol’s iodine and polyvinylpyrrolidone-iodine (66–68). As a result, immunomodulators must be used as an alternative therapy for bovine reproductive problems.

### Immunomodulators role in the treatment of infertility

Extensive research work has been carried for the treatment of metritis and endometritis with immunomodulators in cattle as well as in the mares. Immunomodulators’ role in uterine defense systems is discussed below.

### Reproductive hormones used as an immunomodulator in the treatment of uterine infection

The pathogenicity of microorganisms present in the post-partum uterus at all stages of estrus is regulated by the cyclical pattern of steroid hormone concentration. The endometrium is more susceptible to infection under progesterone dominance than estrogen dominance, as evidenced by increased blood flow to the uterus, increased mucus production, and increased PMN activity during the estrogen phase of the ovarian cycle, but reduced bacterial permeability in endometrial epithelial delays leukocyte stimulation during the luteal phase (56). Chacin et al. (69) observed that when animals were exposed to estrogen, their PMN activity increased. Progesterone exhorts an inhibitory action on the myometrial contraction than estradiol because progesterone inhibits the electrical conductivity in the myometrium (70). As a result, debris clearance through an open cervix is ineffective (71). Besides these two hormones, PGF_2α_ that primarily synthesized in the caruncular endometrium of the uterus can contract and clear the uterine debris (72). Oxytocin causes PGF2 secretion from the uterus, which stimulates oxytocin release from the corpus luteum, which ultimately induces PGF2 secretion from the endometrium. Until luteolysis is complete, this positive feedback process operates (73). As a result, from day 16 of the estrous cycle to oestrus, peripheral blood progesterone decreases. Involution is delayed if the length of PGF2 release postpartum is too short; if the interval is prolonged, the involution process is expedited (74). After normal calving, PGF2 metabolite concentrations in the peripheral blood are high and do not recover to baseline until 14 days after calving (72). Conversely, metritis or acute endometritis in cows caused by uterine microbe infection obscures involution (75).

As a result, there are three reasons to treat endometritis with PGF2. Exogenous prostaglandin therapy administered to calves with a functional corpus luteum causes luteolysis and causes the animal to go into heat, lowering progesterone’s inhibitory effect on the uterine defense system or boosting it with estrogen. Myometrial contractions are also caused by PGF_2_ (76) which may be a mechanism that expels debris and micro-organisms that contaminate the uterine lumen after calving. Third, PGF2 may have a stimulatory effect on uterine PMN phagocytic activity (77). As a result, endometritis in cows with a functional corpus luteum has been treated using PGF2’s luteolytic activity.

### *Escherichia coli* lipopolysaccharides

PMNs are thought to be attracted to the uterine lumen by the chemoattractant effect of *E. coli* lipopolysaccharides (LPS). This increase in PMNs in the endometrium could help cows and mares recover from endometritis (78). Targowski (79) found that an intrauterine infusion of 100 g *E. coli* LPS increased the number of PMNs found in uterine secretions at 6 and 24 h after treatment in healthy cows. Rashid Dar et al. (7) evaluated the immunomodulatory effect of curcumin 30 M, lipopolysaccharide (LPS), and/or flagellin (100 ng/mL each) on prostaglandin E2 (PGE2) and proinflammatory cytokines (PIC) production using primary bubaline endometrial stromal cells. They discovered that LPS stimulates PGE2 production strongly, but flagellin has a lesser stimulatory impact. LPS significantly boosted the transcripts of IL8 and IL6 in bubaline endometrial stromal cells when compared to IL1 and TNF. Curcumin prevented the LPS-induced upregulation of PIC while significantly lowering IL8 levels. Curcumin’s inhibitory effects on inflammatory mediators suggest that it could be used to treat bovine endometritis.

### Oyster glycogen

Oyster Glycogen originally synthesized by animal cells for energy storage is a polymer and has immunomodulatory action (80). It has the property to attract PMN cells into the uterine lumen (81). In healthy cows, PMN migration into the uterine lumen is accelerated after intrauterine administration of oyster glycogen (OG), with neutrophils accounting for up to 90% of all cells identified in uterine secretions (82). OG doses ranging from 0.1 to 10% in 60 mL of vehicle produced similar results, with a peak in PMN level 12 h after treatment (81). There were detectable IgG concentrations in uterine secretions after therapy, but no IgA; ovariectomized cows had similar results after exogenous progesterone and estrogen injections (69).

10 mg/mL instilled intrauterine administration of oyster glycogen (OG) to cure subclinical endometritis in repeat breeding crossbred cows to earlier reports (80). Although the recovery rate was higher (*p* < 0.05) than the control (16.7%), there was no significant difference in the conception rate between the treated and control groups. At 24 h after treatment, the treated cows had significantly higher TLC (*P*0.01) than the control cows. From the day of treatment, the infusion of OG resulted in a significant (*p* < 0.05) decrease in plasma levels of inflammatory mediators (LPO and NO; 71.58 ± 2.57, 76.67 ± 4.59 μmol/L) to subsequent estrus (60.00 ± 2.06, 60.08 ± 2.17 μmol/L), respectively. Therefore, treatment with OG can resolve not only the subclinical endometritis in repeat breeder crossbred cows but also reduces the chances of oxidative stress in the blood plasma due to its immunomodulatory effect. Similar studies has been carried out on endometritic cows to determine the immunomodulatory effect of oyster glycogen (83). They administered estrus cows 500 mg of oyster glycogen in phosphate-buffered saline intrauterine. The Polymorph nuclear (PMN) cell count in uterine fluid increased significantly (13.14 ± 1.35 vs. 73.71 ± 3.59) before and after 24 h of treatment. After the first post-treatment insemination, they found that cows with endometritis had a 60% conception rate, compared to 70% in normal cows.

### Leukotriene B4

The arachidonic acid metabolite leukotriene B4 (LT B4) is a potent chemoattractant that can induce the migration of Polymorph nuclear cells into the bovine uterine lumen. A single infusion of 30 nmol/L Leukotriene B4 can raise the amount of intrauterine Polymorph nuclear cells by up to 5–10 times in 24 h (84). PMNs composed 25–30% of the total leukocyte count before stimulation; after therapy, they made up 85% of the total leukocyte count (85). For the treatment of subclinical endometritis in recurrent breeding crossbred cows, Krishnan et al. (80) infused 50 mL of 30 nmol/L Leukotriene B4 (LTB4) intrauterine. The recovery rate was higher (83.3%; *p* < 0.05) than control (16.7%). The treated group saw a non-significant increase in conception rate when compared to the control group. When compared to control cows, treated cows showed higher TLC (P0.01) 24 h after treatment. From the day of therapy, LTB4 infusion resulted in a significant (*p* < 0.05) decrease in plasma levels of inflammatory mediators (LPO and NO; 667.15 ± 42.85, 753.73 ± 32.78 nmol/L) to subsequent estrus (555.56 ± 42.53, 630.88 ± 31.16 nmol/L), respectively. They reported that giving LTB4 to repeat breeding crossbred cows heals subclinical endometritis and reduces oxidative stress, as seen by lower plasma LPO and NO levels after immunomodulatory treatment.

### Plant-based immunomodulators

Several plant-based immunomodulators are available to treat reproductive disorders in bovines. The immunomodulatory effect is due to the presence of the active ingredient in these plants. The active ingredients like allicin in garlic (86), *sitoindosides VII-X* and *withaferin in ashwagandha* (87), hydroalcoholic and hydroacetonic in neem (88) and eugenol in tulsi (89) are some examples that have antibacterial and antifungal property. The immunomodulatory properties of various plants are now being studied. Endometritis has traditionally been treated with *Cordifolia*. Endometriotic cows were treated with 50 mL (3,000 mg total dose) of aqueous extracts of *Tinospora cordifolia* for 3 days and saw a 66.67 per cent recovery rate and a 27.27 percent conception rate, respectively (90). New antifungal substances, such as *Rosemarinus officinalis* and *Thymus vulgaris*, were found to have a good therapeutic impact, as well as an immunostimulant and free radical scavenger capabilities, against mycotic endometritis caused by *Candida albicans* (91). Other plants like Neem have been used for their immunomodulatory and therapeutic activity against endometritis (88). Extracts of 30 mL hydro-alcoholic Neem bark and hydro-acetonic Neem bark were given intra-uterine for 7 days beginning on the day of estrus. Although clinical recovery and improved conception were reported from both groups, it was concluded that hydro-alcoholic extract of Neem had better therapeutic value. Some immunomodulatory herbs, such as *Withenia somnifera*, are known to have a gonadotropic effect in animals, raising gonadal weight by increasing female follicle size and male seminiferous tube cell layers (92). *Withenia somnifera* improves spermatogenic activity may be due to supporting the hypothalamic-hypophysial-gonadal hormonal axis and testosterone balance in testes (92). Also, *Withenia somnifera* increases testosterone and progesterone concentration in male rats and decreases triglyceride and cholesterol in both male and female rats (93) (Table 2).

**Table 2:** Different immunomodulatory herbs and their action used in various reproductive disorders.

Immunomodulatory herb	Action	Disease	References
*Allium sativum* (Garlic)	Antimicrobial	Endometritis	(86)
*Azadirachta indica* (Neem)	Antimicrobial, antiviral, antifungal		(94)
*Aristolochia indica* (Isharmur)	prevent uterine infection by augmenting the local immune system		(95)
*Ocimum sanctum* (Tulsi)	Analgesic, antiasthmatic, antimicrobial, antifungal		(96)
*Curcuma longa* (Turmeric)	Antibacterial		(97)
*Angelica, Talcum, Radix astragali, Rehmannia root, Tuckahoe, Peach kernel,*	Increases myometrium contraction	Retention of fetal membrane	(98)
*Wallichii, Angelica, Garden balsam, Rhizoma ligustici, Radix codonopsitis, Motherwort*			(99)
*Notopterygium root, Fructus meliae toosendan, Radix bupleuri, Semen litchi, Fennel Frankincense*			(100)
*Lepidium sativum* (asalio), *Pennisetum americanum* (pearl millet) *Anethum graveolens* (suva), *Trigonella foenum-graecum* (methi) seeds,			(101)
*Bonducella* (kanarej) and *Caesalpinia*			(95)
Oil of seed of *Brassica napus*, Gum of *Acacia nilotica subsp. Indica*, root of *Ficus benghalensis,* dried flower of *Corchorus capsularis, Basella alba* leaf paste, *Boerhavia diffusa* whole plant,			(102)
*Hedychium spicatum* (seeds), *Girardinia diversifolia* (dried leaves),		Smooth Delivery	(103)
*Citrus medica* (fruit juice) is mixed with *Cuminum cyminum* (powdered fruits) *or Gomphrena serrata* (whole plant paste)		Cervico Vaginal Prolapse	(104)
*Pedalium murex* (fruits), *Convolus microphyllus* (roots), *Cicer arietimun* (germinated Bengal gram),		Repeat Breeder	(105)
Seed*s* of *Nigella sativa* and Roots of *Abroma augusta*	Increases the expression of certain enzymes like ovarian glucose-6 dehydrogenase and 3 beta HSD, advances the onset of puberty, enhances the number of medium, large-sized follicles and embedded follicles, also induces oestrus and ovulation	Follicular Dynamics	(106)
Root of *Urtica dioica and* Leaf of *Murraya koenigii*			(107)
*Trigonella foenum-graecum*	Increases serum estradiol hormone level		(108)
*Trigonella foenum-graecum (Fenugreek)*	Active ingredients like saponins and alkaloids found in the seed oil of *Fenugreek* stimulates ovarian activity and oviduct	Anestrus	(109, 110)
*Asparagus racemosus*	Estrogenic property can stimulate ovarian function and uterine tonicity		(111)
Leaves of *Murraya koenigii* (Curry leaves) and *Aegele marmelos* (Bael leaves)	Rich source of antioxidant minerals (Co, Cu, Fe, I, Mn, Se, Zn) and Vitamin (Vit A, B, C E)		(112)
Fruits of *Semecarpus anacardium* (bhilama) mixed with Silk cotton leaves powdered, fermented boiled rice water and *Pergularia daemia* pods (dudheli)			(113)
*Epimedium sagittatum* (Horny goat weed)	Enhance semen volume, sperm motility, concentration, testosterone levels and also act as an aphrodisiac	Semen Production	(114)
*Tinospora cordifolia*	Improve semen quality, libido and semen antioxidant profile in bucks		(115)
*Leuzea carthamoides and Eurycoma longifolia*	Semen quality and libido have been improved in boars through feeding a mixture of these plant		(116)

## Conclusion

Reproductive failure is the major cause of infertility and has been identified as a disease. The antigens of reproductive hormones, spermatozoa and ova can all contribute to the development of antibodies that can interfere with the hormones’ receptors, immobilize spermatozoa and ultimately result in immunoinfertility. This results in anovulation, delayed ovulation, failure of fertilization, prolonged uterine involution, extended calving interval, prolonged post-partum estrus and reduced conception rate. The level of these antibodies can subside into the blood by giving sexual rest to the female animals, by applying assisted reproductive technologies and by treating the animals with immunomodulators like LPS, Oyster glycogen, etc.

## Author contributions

VG: conceptualization and drafting. TM: conceptualization and review. MB: review and editing. RD: drafting, review, and editing. RK: editing. DN: review and editing. MSe, RR, SD, NS, and MSi: review. All authors contributed to the article and approved the submitted version.

## Conflict of interest

The authors declare that the research was conducted in the absence of any commercial or financial relationships that could be construed as a potential conflict of interest.

## Publisher’s note

All claims expressed in this article are solely those of the authors and do not necessarily represent those of their affiliated organizations, or those of the publisher, the editors and the reviewers. Any product that may be evaluated in this article, or claim that may be made by its manufacturer, is not guaranteed or endorsed by the publisher.
